# High-Resolution Ultrasound Spectroscopy for the Determination of Phospholipid Transitions in Liposomal Dispersions

**DOI:** 10.3390/pharmaceutics14030668

**Published:** 2022-03-18

**Authors:** Diego Romano Perinelli, Marco Cespi, Giovanni Filippo Palmieri, Annalisa Aluigi, Giulia Bonacucina

**Affiliations:** 1 Chemistry Interdisciplinary Project (CHIP), School of Pharmacy, Via Madonna delle Carceri, University of Camerino, 62032 Camerino, Italy; diego.perinelli@unicam.it (D.R.P.); marco.cespi@unicam.it (M.C.); gianfilippo.palmieri@unicam.it (G.F.P.); 2 Department of Biomolecular Sciences, University of Urbino Carlo Bo, Piazza del Rinascimento, 6, 61029 Urbino, Italy; annalisa.aluigi@uniurb.it

**Keywords:** main transition, bilayer, loaded liposomes, elution, encapsulation

## Abstract

High-resolution ultrasound spectroscopy (HR-US) is a spectroscopic technique using ultrasound waves at high frequencies to investigate the structural properties of dispersed materials. This technique is able to monitor the variation of ultrasound parameters (sound speed and attenuation) due to the interaction of ultrasound waves with samples as a function of temperature and concentration. Despite being employed for the characterization of several colloidal systems, there is a lack in the literature regarding the comparison between the potential of HR-US for the determination of phospholipid thermal transitions and that of other common techniques both for loaded or unloaded liposomes. Thermal transitions of liposomes composed of pure phospholipids (dimyristoylphosphatidylcholine, DMPC; dipalmitoylphosphatidylcholine, DPPC and distearoylphosphatidylcholine, DSPC), cholesterol and their mixtures were investigated by HR-US in comparison to the most commonly employed microcalorimetry (mDSC) and dynamic light scattering (DLS). Moreover, tramadol hydrochloride, caffeine or miconazole nitrate as model drugs were loaded in DPPC liposomes to study the effect of their incorporation on thermal properties of a phospholipid bilayer. HR-US provided the determination of phospholipid sol-gel transition temperatures from both attenuation and sound speed that are comparable to those calculated by mDSC and DLS techniques for all analysed liposomal dispersions, both loaded and unloaded. Therefore, HR-US is proposed here as an alternative technique to determine the transition temperature of phospholipid membrane in liposomes.

## 1. Introduction

Liposomes are phospholipid-based multi- or unilamellar nanovesicles of great interest as drug delivery carriers [1]. Over the years, liposomes were employed to successfully encapsulate drugs or active ingredients in order to improve their stability and enhance efficacy and reduce the related toxicity in vivo. As such, some liposomal formulations containing anticancer drugs or biotherapeutics have reached the market and many others are under clinical or pre-clinical investigations [2,3,4]. The peculiar structural properties influencing the in vitro/in vivo behaviour of liposomes are mainly related to lipid composition. Phospholipids, which are the main component, display characteristic thermal transitions, affecting the permeability of the bilayer and consequently the applicability of liposomes in drug delivery both in terms of encapsulation, targeting and release properties. Specifically, the permeability of the liposomal membrane increases from an ordered gel phase to a disordered fluid phase as occurs above the main phase transition temperature (Tm) of phospholipids [5].

The traditional technique for determining lipid phase transitions is differential scanning calorimetry (DSC), which measures the variation of heat flow as a function of temperature due to the heat absorption occurring when phospholipids undergo the main transition [6]. Although calorimetric parameters related to the transition can be calculated from DSC (e.g., enthalpy), other techniques could also provide chemical–physical information for a thorough investigation of lipid transitions in liposomal formulations. One of them is high-resolution ultrasound spectroscopy (HR-US). It is a spectroscopic technique, which employs sound pressure waves with a frequency above the audible range for humans (~20 kHz) to study structural properties of a material in a fast, non-destructive and reliable manner [7]. Specifically, this technique measures the variation of ultrasound parameters (sound speed and attenuation) when high-intensity ultrasound waves travel through the sample [8]. Being particularly sensitive to the structural changes in the material as a function of temperature and concentration, this technique has been successfully employed for the characterization of plenty of colloidal systems, investigated for their applications in pharmaceutical and biomedical fields [9,10]. Despite HR-US already being employed in some works for the characterization of the temperature-dependent behaviour (e.g., gel-to-sol transition) of liposomes composed of a single phospholipid [11] or a mixture of phospholipids and other components [12,13], a general overview of HR-US and liposomes characterization over temperature lacks in the literature. Specifically, there are few reports focusing on the direct comparison of HR-US results with those from the commonly used calorimetric technique both on unloaded and drug-loaded liposomes. Thus, in this work, HR-US is presented as a complementary technique for the determination of phospholipid main transition in liposomes in comparison to the most commonly employed calorimetry. Particularly, the main transition and thermal behaviour of liposomes prepared with pure phospholipids (DMPC, DPPC and DSPC), their mixtures and phospholipids in the presence of cholesterol were investigated. In addition, three model drugs with different solubility in water (tramadol hydrochloride, caffeine and miconazole nitrate) were loaded in liposomes to study the effect of their incorporation in terms of the thermal behaviour.

## 2. Materials and Methods

### 2.1. Materials

**Scheme 1:**
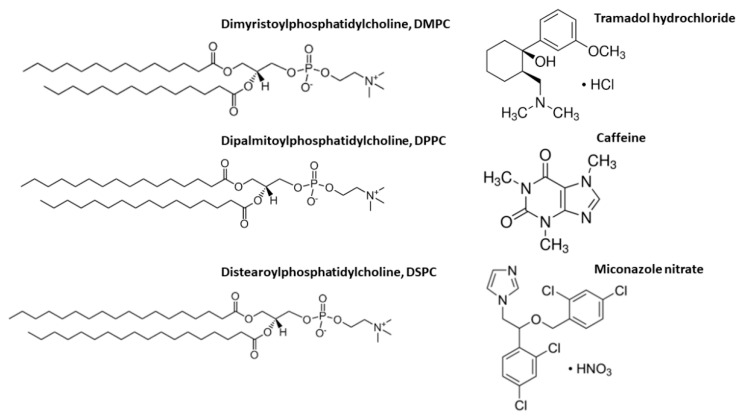
Chemical structures of phospholipids (DMPC, DPPC and DSPC) and drugs (tramadol hydrochloride, caffeine, miconazole nitrate) used in this study.

All phospholipids (dimyristoylphosphatidylcholine, DMPC; dipalmitoylphosphatidylcholine, DPPC and distearoylphosphatidylcholine, DSPC) were purchased from Avanti polar lipids, inc. (Alabaster, AL, USA). Cholesterol and anhydrous caffeine (molecular weight: 194.19 Da) were purchased from A.C.E.F. (Fiorenzuola D’Arda, Italy). Tramadol hydrochloride (molecular weight: 299.83 Da) and miconazole nitrate (molecular weight: 479.1 Da) were generously donated by Janssen-Cilag SpA Co. (Latina, Italy). The chemical structures of phospholipids and drugs used in this study are reported in Scheme 1.

### 2.2. Methods

#### 2.2.1. Liposomal Dispersion Preparation

All liposomal dispersions were prepared by thin-film hydration method. The required amounts of phospholipids (DMPC, DPPC and DSPC) and cholesterol were dissolved in chloroform, then dried under vacuum and rehydrated with ultrapure water or 50 mM sodium phosphate buffer pH 7.4 at a temperature above the sol-gel transition of each phospholipid. After hydration, the liposomal dispersions were sonicated at a temperature above the sol-gel transition using a probe sonicator (Ultrasonic cell disruptor, Microson XL2000, Farmingdale, NY, USA) at 60 W for three cycles of 2 min each. Liposomal dispersions were stored at 4 °C for further use. The following formulations were prepared: (a) DMPC, DPPC or DSPC liposomal dispersions at three concentrations (2 mg/mL, 5 mg/mL and 10 mg/mL); (b) their mixture at 1:1 weight ratio (final concentration 10 mg/mL); (c) DMPC, DPPC or DSPC liposomal dispersions at a concentration of 10 mg/mL in the presence of 10% and 20% of cholesterol (with the respect to the moles of phospholipids). Moreover, the DPPC liposomal dispersions at the concentration of 10 mg/mL were loaded with three model drugs: caffeine, tramadol hydrochloride and miconazole nitrate at a molar ratio 5:1 with the respect to the moles of phospholipids. The un-encapsulated drug was removed by gel filtration chromatography using pre-packed disposable PD-10 Desalting Columns containing 8.3 mL of Sephadex™ G-25 Medium (GE Healthcare Bio-Sciences AB, Sweden) according to the manufacturer instructions for the gravity protocol [14]. The amount of the un-encapsulated drug was determined spectrophotometrically (UV-1800 spectrophotometer, Shimadzu, Japan) by building up a calibration curve in water for tramadol hydrochloride (y = 5.8519 x + 0.0136; R2 = 0.999) at the wavelength of 271 nm and in methanol for caffeine (y = 41.399 x + 0.0139; R2 = 0.999) and for miconazole nitrate (y = 0.048 x + 0.0092; R2 = 0.999) at the wavelength of 272 nm and 226 nm, respectively. The encapsulation efficiency % (EE %) was calculated according to the following equation:

(1) $$EE\;\%=\frac{Added\;amount\;of\;the\;drug-unencapsulated\;drug}{Added\;amount\;of\;the\;drug}\times 100$$

#### 2.2.2. Microcalorimetry (mDSC)

Calorimetric scans were collected using a microDSC III (Setaram, France). 0.750 g of the liposome dispersions and the same amount of ultrapure water or sodium phosphate buffer 50 mM pH 7.4 were filled in Hallostey calorimetric cells before starting the analysis. Sample and reference were subjected to a heating and a cooling ramp from 5 °C to 80 °C at 1 °C/min after equilibration at 5 °C for 20 min. The temperature (Tm, °C) and enthalpy (ΔH, J/g of solution) associated with the transitions were calculated from the peak and the area of the thermograms, respectively, by using the software of the instrument (Setsoft2000, Setaram, France) through the tangent method. All measurements were performed in triplicate.

#### 2.2.3. High-Sensitive Ultrasound Spectroscopy (HR-US)

Ultrasonic analyses were carried out using an HR-US 102 high-resolution spectrometer (Ultrasonic Scientific, Dublin, Ireland) operating at the frequency of 5.4 MHz. The two ultrasonic cells were filled with the liposomal dispersion and ultrapure water or 50 mM sodium phosphate buffer pH 7.4 as reference media and equilibrated at 5 °C for at least 20 min. Then, the variation of the ultrasound parameters attenuation and sound speed was collected as a function of temperature by applying the same thermal programme reported for mDSC analyses (Section 2.2.2). Temperature was controlled using a HAAKE C25P thermostat. Ultrasonic attenuation and sound speed are reported as differential relative values, obtained by subtracting the contribution of the reference medium (ultrapure water or phosphate buffer) from the value recorded in the sample cell. Transition temperatures were calculated by the peak value obtained from the attenuation profile or the minimum of the first-derivate of the signal in the case of sound speed [9]. All measurements were performed in triplicate.

#### 2.2.4. Dynamic Light Scattering (DLS)

DLS analyses were performed using a Malvern Zetasizer nanoS (Malvern instrument Worcestershire, Malvern, UK), detecting the scattered light at 173°. The hydrodynamic diameter of liposomes, measured at 25 °C after an equilibration time of 180 s, was reported as Z-average value and particle size distribution was expressed as polydispersity index (PDI). The scattered intensity (counts) of liposomal dispersions, at a fixed position (4.65) and attenuation (11), was also recorded in the range of temperature from 5 °C to 80 °C with a temperature step of 1 °C. The transition temperature was calculated from the Boltzmann regression curve as reported by Michel et al. [15] according to the equation:

(2) y=A1−A21+ex−x0Δx

where *A*_1_ and *A*_2_ are the plateaux of the curve, *x*_0_ is the *x* value of the curve at the half distance between the plateau and Δ*x* is the width of the slope. All analyses were performed in triplicate.

## 3. Results

### 3.1. Particle Size Determination of the Liposomal Dispersions

All the liposomal dispersions prepared in ultrapure water at a concentration of 10 mg/mL have comparable particle size distributions after ultrasonication with Z-average values between 80–140 nm and a polidispersity index (PDI) between 0.20–0.33 (Table 1). In detail, Z-average and PDI values slightly increased, moving from DMPC to DPPC and DSPC liposomes and for liposomes in the presence of cholesterol. Moreover, independently from the concentration of phospholipids (2 mg/mL, 5 mg/mL and 10 mg/mL), the applied ultrasonication conditions were able to reduce the size of the phospholipid vesicles in the nanometric range and to achieve a low polidispersity (Table S1). DPPC liposomes prepared at 10 mg/mL in 50 mM sodium phosphate buffer pH 7.4 have a similar particle size to those prepared in ultrapure water and the encapsulation of the drugs (tramadol hydrochloride, caffeine or miconazole nitrate) determined only a slight increase in Z-average and PDI values (Table 1).

### 3.2. Characterization of Single-Phospholipid Liposomal Dispersions

**Figure 1:**
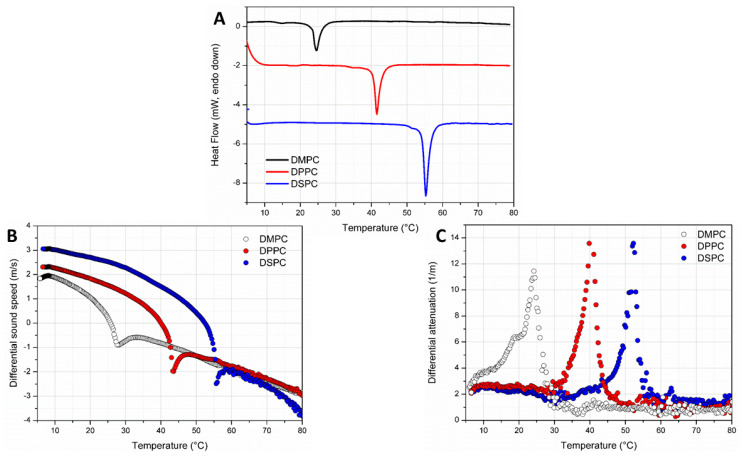
mDSC traces (**A**), variation of sound speed (**B**) and variation of attenuation (**C**) over temperature from HR-US for pure phospholipids (DMPC, DPPC, DSPC).

mDSC traces showed the characteristic endothermic event associated with the sol-gel main transition of a single phospholipid bilayer (~24 °C for DMPC, ~41 °C for DPPC and ~54 °C for DSPC) (Figure 1A and Figure S1). The area related to the transition (J/g of solution) is proportional to the concentration of the liposomal dispersions (2 mg/mL, 5 mg/mL and 10 mg/mL), from which the enthalpy can be calculated. At the same concentration of phospholipid, the enthalpy associated with the transition is also dependent on the acyl chain length of phospholipids, being increased moving from DMPC, DPPC to DSPC (Table 2 and Table S2) [16].

mDSC traces were compared to the result obtained as a function of temperature by HR-US. Indeed, Figure 1B shows the variation of the ultrasound parameter sound speed over the temperature recorded for the liposomal dispersions prepared using DMPC, DPPC, DSPC at 10 mg/mL. Sound speed decreases over temperature but a marked inflexion point was observed near the Tm of phospholipids. As regards the other ultrasonic parameter, attenuation has an intrinsic low dependency on temperature than sound speed, but it steeply deviates from baseline, reaching a maximum value in the nearby of the Tm transition and then decreases, returning to the baseline values. Generally, the baseline values above the phase transition temperature are lower than those below the transition itself and tend to decrease slightly at the temperatures at which the thermal process can be considered complete. The effect of the liposomes’ concentration (2 mg/mL, 5 mg/mL and 10 mg/mL) is evident from both sound speed and attenuation profiles. As previously reported [17], an increase in liposomes concentration determines a decrease in the calculated slope value of the sound speed signal and slightly higher attenuation values of the baseline both before and after the transition (Figure S2). The transition values from HR-US traces, calculated from the minimum of the first derivative of raw sound speed data (Figure S3) or from the maximum value of attenuation, are consistent with the literature and comparable to those calculated from mDSC (Table 2) [5]. A good agreement in term of Tm, determined by calorimetry and HR-US, was also found for liposomal dispersions prepared with DMPC, DPPC and DSPC at the other analysed concentrations (2 mg/mL and 5 mg/mL) (Table S2).

### 3.3. Characterization of Mixed-Phospholipid Liposomal Dispersions

**Figure 2:**
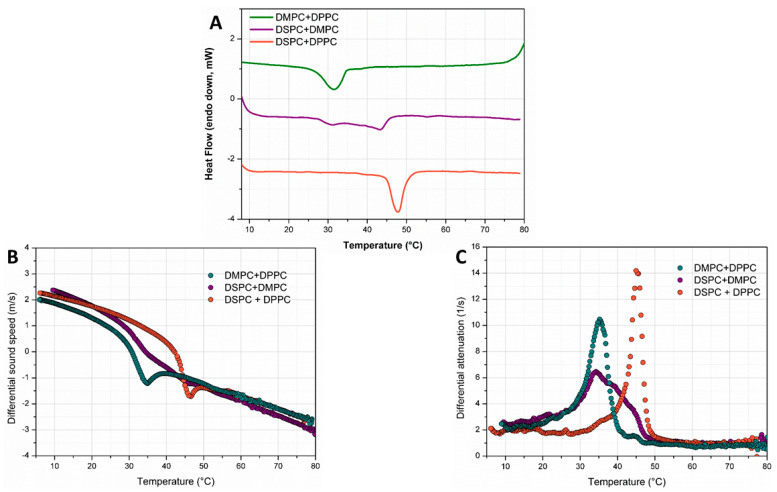
mDSC traces (**A**), variation of sound speed (**B**) and variation of attenuation (**C**) over temperature from HR-US for binary mixture (1:1) of phospholipids (DMPC/DPPC, DPPC/DSPC and DMPC/DSPC).

The ability of HR-US technique to detect sol-gel transitions of phospholipids was also evaluated for liposomal dispersions formed by a binary mixture of DMPC, DPPC or DSPC at a weight ratio 1:1. As reported in the literature [18,19,20], the binary mixture DMPC/DPPC and DPPC/DSPC at the analysed ratio show by mDSC a single endothermic transition, broader than and with a maximum (~31 °C and ~47 °C for DMPC/DPPC and DPPC/DSPC mixture, respectively) between that of pure single phospholipids. The mixture of DMPC/DSPC showed instead two defined endothermic transitions at ~29 °C and 42 °C in the gel-liquid coexistence region due to a larger difference in alkyl chain length of phospholipids composing the liposomal membranes (C14 for DMPC and C18 for DSPC) (Figure 2A). This behaviour can be evidenced also by HR-US analysis following sound speed or attenuation variation over temperature. Indeed, a steeper decrease of sound speed was observed for the mixtures composed of DMPC/DPPC and DPPC/DSPC, while a more flattened profile in the range of temperature of the transition was observed for the mixture DSPC/DMPC (Figure 2B). This is also reflected in the broadness of the attenuation profile for DSPC +DMPC mixtures in comparison to DMPC + DPPC and DSPC + DPPC (Figure 2C).

### 3.4. Characterization of Phospholipid Liposomal Dispersions in Presence of Cholesterol

**Figure 3:**
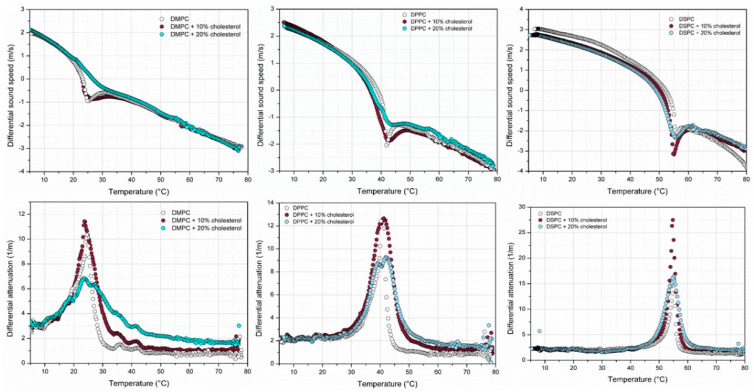
Variation of sound speed and attenuation for DMPC, DPPC and DSPC in the presence of different percentages of cholesterol (0%, 10% and 20% with the respect to moles of phospholipids).

Figure 3 reports the effect of different percentages of cholesterol (10% and 20% with the respect to the moles of phospholipids) on the sound speed and attenuation ultrasound parameters recorded in the range of temperature 5–80 °C. The stabilizing effect exerted by cholesterol on the gel-to sol transition of phospholipid is evident from both sound speed and attenuation traces. In sound speed traces, the liposomal dispersions prepared in the presence of cholesterol showed a less pronounced inflexion of the signal in the proximity of the transition temperature. On the other side, the attenuation peaks are broader for the liposomal dispersions prepared in the presence of cholesterol and the maximum variation of attenuation in the proximity of the transition temperature was much lower for the sample containing cholesterol at 20%. However, the presence of the cholesterol at the two tested concentrations did not suppress completely the sol-gel transition, as also confirmed from mDSC traces reported in Figure S4. Specifically, the effect of cholesterol on the transition temperature of liposomal dispersions is more pronounced in the order DMPC > DPPC > DSPC and it is probably related to the acyl chain length of phospholipids. Shorter acyl chains promote the condensing effect of cholesterol on phospholipid bilayer in which lipid acyl chains are more ordered and oriented perpendicularly to the bilayer plane [21,22].

### 3.5. Thermal Transition of Phospholipids by DLS Analysis

**Figure 4:**
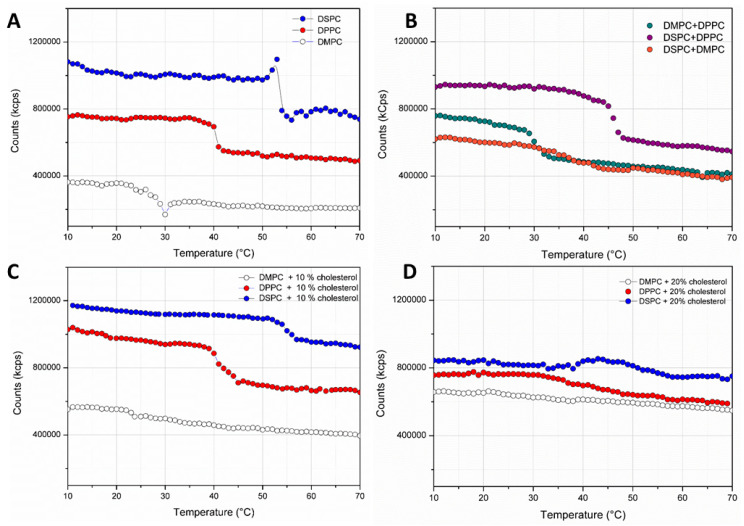
Variation of counts (kcps) from DLS over temperature for pure phospholipids (DMPC, DPPC and DSPC) (**A**), binary mixtures (1:1) of phospholipids (DMPC/DPPC, DPPC/DSPC and DMPC/DSPC) (**B**) and phospholipids in the presence of 10% (**C**) or 20% of cholesterol (with the respect to moles of phospholipids) (**D**).

DLS is a technique also employable for the determination of the thermal gel-to sol transition of phospholipids by analysing the variation of the raw scattered light from the sample over temperature [12,15]. Scattered light intensity depends also on the variation of the refractive index of the sample in addition to the size and number of particles in dispersions. A change in the refractive index of the liposomal membrane occurs near gel-to-sol transition as a consequence to the decreased density of the bilayer and leading to a lowering in the scattered light intensity to the detector (kilo counts per second, kCps) [23]. For pure phospholipids, a stepwise decrease of the counts to the detector occurs in the proximity of transition. The transition temperatures calculated are reported in Table 2 and are comparable with those calculated from mDSC and HR-US. Notably, the total counts seem to be dependent on the molecular weight of phospholipids since they increase in the order DMPC > DPPC > DSPC for the analysed liposomal dispersions. The height of the step related to the count variation in the proximity of the transition is also found dependent on the MW of the phospholipid used. Indeed, it is more pronounced for DSPC liposomes (Δkcps ~306,000) with the respect to DPPC (Δkcps ~120,000) and DMPC liposomes (Δkcps ~54,000). The technique was also able to highlight the gel-to-sol transition on phospholipid mixtures (Table 2). In this case, the steepness of the decrease in counts in the proximity of the transition was lower than that calculated for liposomal dispersions composed of pure phospholipids, reflecting the broadening of the calorimetric peaks (Figure 4A). This is particularly evident for the mixture DSPC/DMPC for which the Δkcps is the lowest and the steepness of the count profile is also much less pronounced, confirming that, as evidenced by mDSC and HR-UR (Figure 4A,B), the sol-to-gel transition spans in a large range of temperatures. The effect exerted by cholesterol on the sol-to-gel transition of pure phospholipids is also noticeable. Indeed, the presence of cholesterol at 10% causes increases in the total counts measured for all liposomal dispersions, suggesting that the density of the bilayer has increased itself. Moreover, the height and the steepness of the variation of counts in the proximity of the transition was also reduced, confirming the stabilizing effect on bilayer exerted by cholesterol (Figure 4C). In the presence of 20% cholesterol, the variation in the measured counts over temperature related to the sol-gel transition is less pronounced with the respect to the samples prepared with 10% of cholesterol and it is negligible for DMPC dispersion (Figure 4D).

### 3.6. Characterization of Phospholipid Liposomal Dispersions in the Presence of Model Drugs with a Different Hydrophobicity

**Figure 5:**
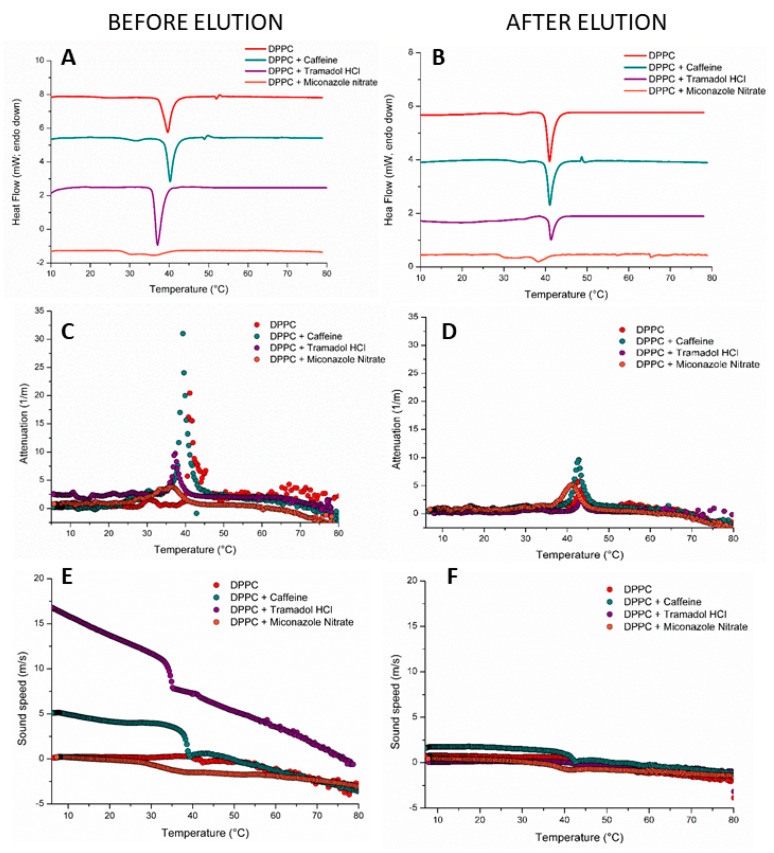
mDSC traces (**A**,**B**) and variation of sound speed (**C**,**D**) and attenuation (**E**,**F**) from HR-US for unloaded or caffeine-, tramadol hydrochloride or miconazole nitrate-loaded DPPC liposomes (10 mg/mL) prepared in 50 mM sodium phosphate buffer pH 7.4 before and after elution.

The effect of the incorporation in DPPC liposomes at the concentration of 10 mg/mL of three model drugs with a different water solubility on the sol-gel transition was investigated. The selected model drugs were tramadol hydrochloride (freely soluble in water) [24], caffeine (sparingly soluble in water at room temperature; solubility ~2 g/100 mL at 20 °C) [25] and miconazole nitrate (practically insoluble in water; solubility ˂0.001 mg/mL) [26]. The calculated EE% of the drugs in DPPC liposomes indicated that the loading was dependent on the different solubility in water of the drug, being the highest for tramadol hydrochloride (EE 7.48%; DPPC to drug molar ratio is 0.375), followed by caffeine (EE 3.66%; DPPC to drug molar ratio is 0.144) and miconazole nitrate (EE 0.45%; DPPC to drug molar ratio is 0.001). mDSC traces and sound speed and attenuation profiles from HR-US of DPPC dispersions were collected before and after removing the free drug to study how the presence of the drug (both encapsulated and un-encapsulated) can affect the sol-gel transition. Firstly, loaded liposomes were prepared in ultrapure water, as for the previously analysed samples; however, the process, performed to separate the un-encapsulated drug according to gel filtration protocol, changed the composition of the media from ultrapure water to that of the buffer used for the elution, making a direct comparison between calorimetric and HR-US profiles recorded before and after the separation process not possible (Figure S5). For this reason, the liposomal dispersions were re-prepared in 0.05 M sodium dihydrogen phosphate and 0.15 M NaCl solution at pH 7.4, the same buffer used for the elution. The preparation in phosphate buffer of DPPC liposomes has a slight effect on the sol-gel transition temperature, determining a decrease of around 1–1.5 °C, and no effect in the enthalpy associated with the transition (Table 2 and Table 3). On the contrary, the elution process itself caused an inherent decrease in the measured enthalpy (J/g of liposome dispersion) due to the 1.4 times dilution of the sample according to the gravity protocol [14]. On the contrary, the presence of the drug exerts relevant effects on transition temperature and enthalpy values both before and after elution (Table 3). Indeed, some relevant differences can be noticed from mDSC traces as well as sound speed and attenuation profiles related to the drug in its free or encapsulated form (Figure 5). For liposomes loaded with tramadol hydrochloride, the transition temperature is shifted, before elution, at a lower value with respect to that of the unloaded liposomes prepared in buffer (33 °C vs 39 °C, respectively). This can be explained considering that tramadol hydrochloride is a salt and the large amount of free ionic species in the media due to the un-encapsulated drug exerting a marked effect on the main transition temperature. Indeed, after elution and the consequent removal in a solution of the un-encapsulated tramadol hydrochloride, the transition temperature increased again up to a value comparable to that of the pure DPPC liposomes. This effect related to the presence of large amounts of free salts on the transition temperature is clearly observable from the mDSC traces and sound speed and attenuation profiles before and after elution. On the other side, the presence of a large amount of free ionic species before elution is confirmed by the much higher sound speed values measured in comparison to those of the same sample after the elution. Differently from tramadol hydrochloride, caffeine is not an ionic drug, and its presence both as an encapsulated or un-encapsulated molecule seems not to affect the transition temperature of DPPC, which remained ~40 °C both before and after dilution. For both liposomes loaded with tramadol hydrochloride or caffeine the broadness of the transition from mDSC (calculated as the difference in the temperature of the onset and the peak) does not change for the samples analysed before and after the elution. On the contrary, the mDSC profiles of DPPC dispersions containing miconazole nitrate—both before and after elution—showed in addition to the shift of the transition at lower temperatures, a marked broadening of the signal, suggesting that the drug is predominantly inserted in the phospholipid bilayer. As reported in the literature for other drugs, the lowering in the transition temperature indicates that the drug interacts with the hydrophobic inner portion of the phospholipid bilayer [27], while the broadening of the transition results from an increase in the ordered state of the alkyl chains of phospholipids [28]. A remarkable decrease in the enthalpy associated with the phospholipid transition was observed for loaded liposomes in comparison to the unloaded ones, suggesting that the loading with any of the model drugs investigated can weaken the molecular interactions between hydrophobic chains of phospholipid molecules and alter the packing of the bilayer itself as observed for other drugs [16,29,30].

## 4. Discussion

High-resolution ultrasound spectroscopy is a useful technique for the characterization of liquid and semi-solid samples in several fields related to material sciences such as food chemistry, agriculture, pharmaceutics and cosmetics [7]. Specifically, this technique has been employed for the investigation of structural and thermal properties of disperse systems such as micelles, emulsions, microemulsions and liposomes, providing reliable results, which can be considered as complementary to those collected by other more commonly employed techniques (e.g., calorimetry) [31,32,33]. These materials can be analysed as a function of concentration or temperature by applying high-intensity ultrasounds at low frequencies generated by a piezoelectric element that converts electricity into mechanical energy. Ultrasonic or acoustic waves propagate longitudinally by a periodic compressional motion (adiabatic compression and decompression). Ultrasound spectroscopy measures the variation of ultrasound wave properties after the interaction with the material, which is related to the energy and the speed of the wave itself. Indeed, a loss of energy (attenuation) or a variation of its velocity of propagation (sound speed) occurs while the ultrasound waves travel into the material. Therefore, ultrasound parameters (attenuation and sound speed) are characteristic of each ultrasound wave. Sound speed is a function of the elasticity and density of the medium as described by the Laplace equation:

(3) $$U=\frac{1}{\sqrt{\beta \rho }}$$

where *ρ* is the density and *β* is the compressibility of the medium, that is the relative change of the medium volume per unit of pressure applied by the ultrasonic wave. Ultrasonic attenuation is a parameter representing the decrease in fluctuation amplitude related to the energy loss occurring as a consequence of the travel of the ultrasound wave through the material. The amplitude of the ultrasound waves and their energy is dissipated since any discontinuities in the material determine ultrasound attenuation. Six different mechanisms contribute to the loss of the ultrasound energy: viscous (α_vis_), thermal (α_th_) scattering (α_sc_), intrinsic (α_int_), structural (α_st_) and electrokinetic (α_elk_).

The total attenuation (α_tot_) is represented by the sum of each contribution:

α_tot_ = α_vis_ + α_th_ + α_sc_ + α_int_ + α_st_ + α_elk_ (4)

Therefore, acoustic attenuation can be described by the following equation:

(5) $$I=I_{0}\;e^{-\mu x}$$

where *I* is the measured intensity of the ultrasound wave thought the material; *I*_0_ is the initial intensity of the ultrasound wave; *x* is the thickness of the material and *µ* is the attenuation coefficient, which is related to the total attenuation (α_tot_). The main phase transition temperature (Tm) of liposomes is defined as the critical temperature at which the phospholipid bilayer changes its physical state from an ordered gel phase (L*_β_*) to a liquid disordered crystalline phase (L_α_). Specifically, phospholipids are fully extended and closely packed at T ˂ Tm, while they are relatively randomly oriented in a short-range disordered phase (T > Tm) [5]. Consequently, this modification of the crystallinity of phospholipid bilayer determines a variation in the compressibility of the medium (*β*) and in the attenuation coefficient (*µ*), mainly related to the intrinsic and scattering components of the total attenuation, which are influenced by density and thickness of the bilayer and size of liposomes. According to this, the main transition of liposomes can be detected using HR-US by following both sound speed and attenuation parameters, since the first one is strongly affected by any variation in compressibility and the second one by any little variations in density. As regards sound speed signal, the increase in the total compressibility determines a typical profile observed for liposomes recorded during a HR-US analysis over temperature. The larger compressibility at temperatures above Tm reflects the higher hydration of liposomal membrane at a sol state with the respect to the gel state, while the minimum in the sound speed values in the nearby of Tm is due to the combined effect related to the increase of heat capacity and isothermal compressibility [34,35]. The increase of attenuation signal, instead, can be ascribed to the higher heterogenicity in the physical state of phospholipid bilayer occurring at temperatures in the proximity of Tm transition in which the bilayer undergoes a conformational change. As reported also in other previous works [12,17,36], HR-US is able to detect the main transition of liposomes with high accuracy in comparison to other commonly employed techniques. As such, the obtained values are comparable to those obtained from the referenced technique such as microcalorimetry and a less common technique for thermal analysis as DLS. In fact, the difference in the calculated Tm values obtained from the three techniques (HR-US, mDSC and DLS) is lower than 1 °C for unloaded and loaded liposomes prepared with a single phospholipid and lower than 2 °C for liposomes prepared with a mixture of phospholipids and in the presence of cholesterol. The little higher discrepancy among the calculated values in these last two cases could be ascribed to a broadening of the experimental signals that affect the determination itself. It is also to be highlighted that HR-US, as well as counts analysis from DLS, is not a technique that measures heat exchange as mDSC from which transition temperatures and energy (i.e., enthalpy) can be calculated, but it responds to molecular and structural events occurring to the material and related to the thermal transition. In this regard, the deviation from the baseline values of attenuation is observed in a range of temperatures broader than that of the heat flow signal from calorimetric analysis, suggesting that conformational changes in the liposomal membrane begin at temperatures lower than the thermal event associated with the main transition. Indeed, for all liposomal dispersions analysed, the width of the attenuation peak spans in a range of temperatures that comprises also the pre-main transition thermal events associated with phospholipid bilayers as the conversion from the flat lamellar gel phase (L_*β*′_) to ripple gel phase (P_*β*′_) [37]. Other studies have investigated the influence of the different hydrophilicity of model drugs in relation to their ability to be located in liposomes such as entrapped inside the aqueous compartment, adsorbed or partially inserted at the level of the water-bilayer interface or partitioned in the phospholipid membrane [38,39]. Specifically, the relationship between the drug localization and the thermal behaviour of loaded liposomes has been mainly investigated by using calorimetry in association with other techniques such as small-angle scattering [40,41]. Here, the comparison between mDSC and HR-US is proposed, instead. Despite the fact that comparable information about the main transition temperatures of phospholipid bilayer can be obtained from both techniques, the partition of hydrophobic drugs into the bilayer can be evidenced as in the presented case of miconazole nitrate by a broadening of the attenuation signal with the respect to that of unloaded liposomes (Figure 5C,D) as well as by the flattening in the sound speed profile (Figure 5E,F). Moreover, HR-US technique is much more sensitive to the presence of un-encapsulated drugs in the medium, as evidenced by the lower attenuation and sound speed values in the samples containing tramadol hydrochloride or caffeine after the elution performed to eliminate the free drug from the medium.

## 5. Conclusions

Ultrasound spectroscopy was shown to be effective in the determination of the temperature-dependent main phase transition of liposomes composed of a single phospholipid, a mixture of two phospholipids and in the presence of cholesterol. Particularly, reliable transition temperatures, comparable to that obtained with classical techniques such as calorimetry or less commonly employed as DLS, were calculated by both analysing sound speed and attenuation parameters from HR-US. Moreover, HR-US was also effective in determining the thermal transitions of liposomes loaded with three model drugs having different hydrophilicity and solubility in water (tramadol hydrochloride, caffeine and miconazole nitrate). The obtained results from HR-US also suggest, as for mDSC, whether drugs are entrapped inside the aqueous compartment or interact with the acyl chains of phospholipid in the liposomal bilayer. This technique can be also sensitive to discriminate the presence of the drug in the samples both as to its free and encapsulated form. Therefore, HR-US configures as an alternative technique to investigate the thermal behaviour of a phospholipid membrane in liposomes.

## Figures and Tables

**Table 1:** Particle size (Z-average; nm) and particle size distribution (PDI) for all unloaded liposome dispersions prepared in ultrapure water at a phospholipid concentration of 10 mg/mL and for all loaded liposomal dispersions prepared in 50 mM sodium phosphate buffer pH 7.4 at a phospholipid concentration of 10 mg/mL.

		Z-Average (nm)	PDI
Liposomes in ultrapure water	DMPC	75.60 ± 3.06	0.224 ± 0.038
	DMPC + 10% Chol	89.55 ± 2.84	0.290 ± 0.046
	DMPC + 20% Chol	96.16 ± 2.08	0.309 ± 0.012
	DPPC	81.13 ± 1.21	0.244 ± 0.009
	DPPC + 10% Chol	101.17 ± 2.19	0.273 ± 0.050
	DPPC + 20% Chol	94.41 ± 4.26	0.328 ± 0.047
	DSPC	101.65 ± 8.31	0.299 ± 0.021
	DSPC + 10% Chol	101.03 ± 3.49	0.304 ± 0.010
	DSPC + 20% Chol	137.87 ± 1.72	0.298 ± 0.029
	DMPC + DPPC	90.29 ± 4.73	0.291 ± 0.060
	DMPC +DSPC	89.71 ± 5.87	0.275 ± 0.008
	DPPC + DSPC	81.76 ± 4.16	0.325 ± 0.052
Liposomes in buffer	DPPC	85.89 ± 6.33	0.264 ± 0.044
	DPPC + Caffeine	103.77 ± 5.13	0.280 ± 0.022
	DPPC + Tramadol HCl	93.23 ± 5.33	0.305 ± 0.055
	DPPC + Miconazole nitrate	82.64 ± 6.38	0.293 ± 0.080

**Table 2:** Thermodynamic parameters (temperature and enthalpy) of the sol-gel transition of liposomes composed of pure phospholipids (DMPC, DPPC, DSPC) at the concentration 10 mg/mL, binary mixtures (DMPC/DPPC, DMPC/DSPC and DPPC/DSPC) and phospholipids in the presence of cholesterol (10% and 20%) as determined by microcalorimetry and HR-US.

	Microcalorimetry			HR-US		DLS
				Attenuation (1/m)	Sound Speed (m/s)	
	Peak (°C)	Onset (°C)	Enthalpy (J/g of Solution)	Temperature (°C)		Temperature (°C)
DMPC	24.63 ± 0.04	23.45 ± 0.04	0.268 ± 0.010	24.27 ± 0.08	24.70 ± 0.53	25.54 ± 0.18
DMPC + 10% Chol	24.71 ± 0.13	23.47 ± 0.01	0.187 ± 0.083	24.60 ± 0.45	23.91 ± 0.23	24.36 ± 0.21
DMPC + 20% Chol	25.28 ± 2.62	18.97 ± 1.07	0.129 ± 0.035	26.47 ± 1.35	24.15 ± 0.32	− ^1^
DPPC	41.60 ± 0.04	40.58 ± 0.04	0.391 ± 0.038	40.42 ± 0.07	40.75 ± 0.06	40.56 ± 0.15
DPPC + 10% Chol	41.13 ± 0.01	39.42 ± 0.17	0.254 ± 0.006	40.97 ± 0.74	39.56 ± 0.08	41.24 ± 0.13
DPPC + 20% Chol	41.23 ± 1.07	37.51 ± 1.33	0.197 ± 0.066	40.61 ± 0.47	40.21 ± 0.10	42.32 ± 1.36
DSPC	54.78 ± 0.15	53.70 ± 0.07	0.631 ± 0.035	52.20 ± 0.08	54.17 ± 0.06	53.85 ± 0.25
DSPC + 10% Chol	54.17 ± 0.98	52.80 ± 0.07	0.484 ± 0.005	52.57 ± 0.14	53.58 ± 0.14	53.69 ± 0.26
DSPC + 20% Chol	53.05 ± 0.08	48.23 ± 1.63	0.433 ± 0.044	51.72 ± 0.38	52.95 ± 0.16	53.68 ± 0.44
DMPC + DPPC	31.59 ± 0.02	27.99 ± 0.16	0.346 ± 0.050	32.94 ± 0.37	32.30 ± 1.48	30.48 ± 0.12
DMPC + DSPC	31.69 ± 0.08 43.32 ± 0.14	28.81 ± 2.47	0.341 ± 0.045	34.72 ± 0.43 ^2^ 43.28 ± 0.37 ^2^	32.50 ± 1.23 43.19 ± 0.65	39.84 ± 3.20
DPPC + DSPC	47.85 ± 0.00	44.79 ± 0.16	0.395 ± 0.018	47.02 ± 0.95	44.45 ± 0.22	45.37 ± 0.19

^1^ Transition temperature for DMPC + 20% Chol sample using DLS was not detectable. ^2^ These transition temperatures were determined by deconvolution analysis (OriginPro 8 software) of the attenuation profile.

**Table 3:** Thermodynamic parameters (temperature and enthalpy) of the sol-gel transition of unloaded and caffeine, tramadol hydrochloride or miconazole nitrate-loaded DPPC liposomes (drug to phospholipid ratio 5:1) prepared in 50 mM sodium phosphate buffer pH 7.4 before and after elution as determined by microcalorimetry and HR-US.

		Microcalorimetry			HR-US	
					Attenuation (1/m)	Sound Speed (m/s)
		Peak (°C)	Onset (°C)	Enthalpy (J/ g of Solution)	Temperature (°C)	
Before Elution	DPPC	39.91 ± 0.41	38.04 ± 0.52	0.401 ± 0.005	39.22 ± 1.04	39.03 ± 0.87
	DPPC + Caffeine 5:1	39.81 ± 0.62	38.39 ± 0.80	0.360 ± 0.063	38.84 ± 0.01	38.94 ± 0.74
	DPPC + Tramadol HCl 5:1	33.75 ± 0.16	32.51 ± 0.01	0.358 ± 0.014	33.20 ± 1.33	34.06 ± 0.25
	DPPC + Miconazole nitrate 5:1	29.86 ± 0.23 36.41 ± 0.96	28.74 ± 1.50	0.212 ± 0.003	35.03 ± 1.20	32.78 ± 0.35
After Elution	DPPC	40.09 ± 0.13	39.07 ± 0.10	0.249 ± 0.007	40.28 ± 1.62	41.27 ± 0.54
	DPPC + Caffeine 5:1	40.18 ± 0.16	39.15 ± 0.06	0.195 ± 0.037	40.41 ± 0.17	42.36 ± 0.32
	DPPC + Tramadol HCl 5:1	40.36 ± 0.03	39.72 ± 0.01	0.156 ± 0.002	40.53 ± 0.04	41.96 ± 0.45
	DPPC + Miconazole nitrate 5:1	30.57 ± 0.24 38.40 ± 0.60	29.41 ± 1.91	0.127 ± 0.016	38.37 ± 0.08	39.10 ± 0.12

## Supplementary Materials

The following supporting information can be downloaded at: https://www.mdpi.com/article/10.3390/pharmaceutics14030668/s1, Table S1: Particle size (Z-average; nm) and particle size distribution (PDI) for all unloaded liposome dispersions prepared in ultrapure water at a phospholipid concentration of 2 mg/mL and 5 mg/mL.; Figure S1: mDSC traces for pure phospholipids (DMPC, DPPC, DSPC) at different concentrations (2 mg/mL, 5 mg/mL and 10 mg/mL); Figure S2: Variation of sound speed and attenuation parameters over temperature for DMPC (A and B), DPPC (C and D) and DSPC (E and F) at different concentrations (2 mg/mL, 5 mg/mL and 10 mg/mL); Table S2: Thermodynamic parameters (temperature and enthalpy) of the sol-gel transition of liposomes composed of pure phospholipids (DMPC, DPPC, DSPC) at the concentration of 10 mg/mL; Figure S3: First derivative of sound speed over temperature for pure phospholipids (DMPC, DPPC, DSPC) at a concentration of 10 mg/mL (A), for binary mixture (1:1) of phospholipids (DMPC/DPPC, DPPC/DSPC and DMPC/DSPC) (B) and for DMPC, DPPC and DSPC in the presence of different percentages of cholesterol (0%, 10% and 20% with the respect to moles of phospholipids) (C); Figure S4: mDSC traces for DMPC, DPPC and DSPC in the presence of different percentages of cholesterol (0%, 10% and 20% with the respect to moles of phospholipids); Figure S5: Variation of sound speed and attenuation parameters over temperatures for DPPC 10 mg/mL, DPPC 10 mg/mL + caffeine (after elution) prepared in water in comparison with the signal obtained by analysing the 50 mM sodium phosphate buffer pH 7.5. All traces were obtained using ultrapure water as reference media for HR-US.
