# Supplementary material for: High-Resolution Ultrasound Spectroscopy for the Determination of Phospholipid Transitions in Liposomal Dispersions
Source: Pharmaceutics. 2022 Mar 18;14(3):668. doi: 10.3390/pharmaceutics14030668 (PMC8955896; doi:10.3390/pharmaceutics14030668)
Supplement: Supplementary file 1 [file pharmaceutics-14-00668-s001.zip › pharmaceutics-1633083-supplementary.pdf]

pharmaceutics

MDPI

Supplementary materials: High-Resolution Ultrasound Spectroscopy for the Determination of Phospholipid Transitions in Liposomal Dispersions

Diego Romano Perinelli, Marco Cespi, Giovanni Filippo Palmieri, Annalisa Aluigi and Giulia Bonacucina

Table S1. Particle size (Z-average; nm) and particle size distribution (PDI) for all unloaded liposome dispersions prepared in ultrapure water at a phospholipid concentration of 2 mg/mL and 5 mg/mL.

	Z-average (nm)	PDI
DMPC 2 mg/ml	122.77 ± 3.71	0.270 ± 0.046
DMPC 5 mg/ml	91.79 ± 8.35	0.330 ± 0.093
DPPC 2 mg/ml	109.67 ± 4.65	0.296 ± 0.036
DPPC 5 mg/mL	92.34 ± 3.40	0.345 ± 0.022
DSPC 2 mg/mL	83.46 ± 5.49	0.368 ± 0.034
DSPC 5 mg/mL	108.07 ± 3.49	0.217 ± 0.012

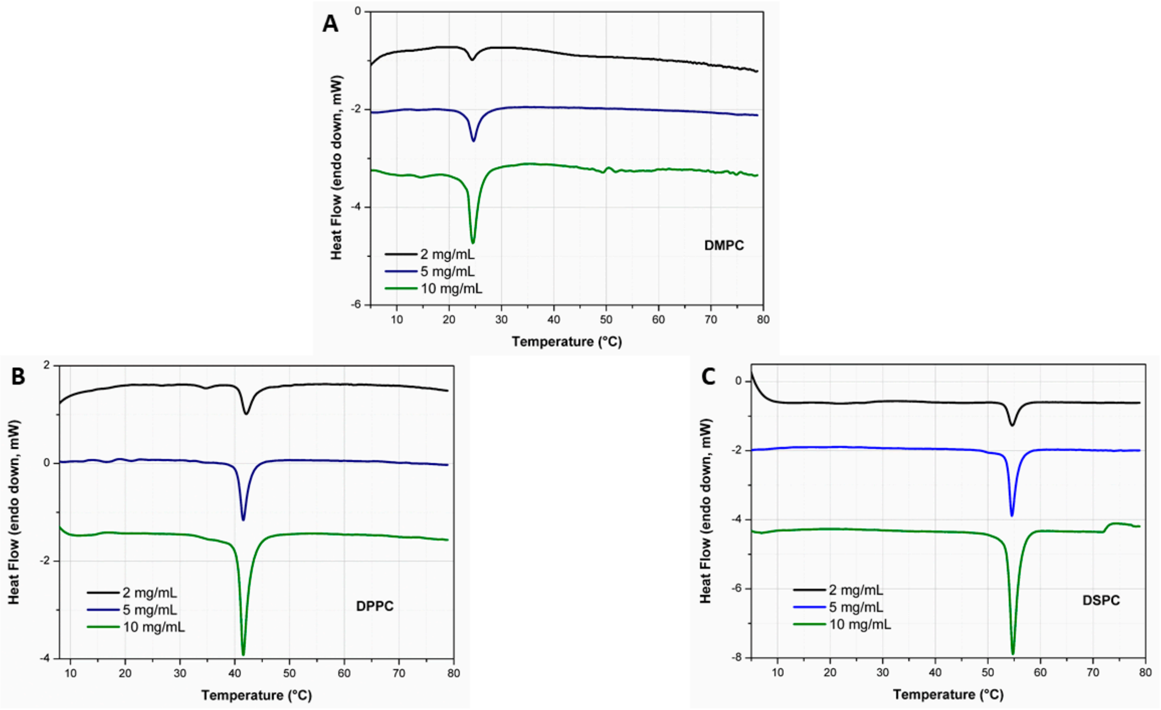

Figure S1. mDSC traces for pure phospholipids (**A**: DMPC, **B**: DPPC, **C**: DSPC) at different concentrations (2 mg/mL, 5 mg/mL and 10 mg/mL).

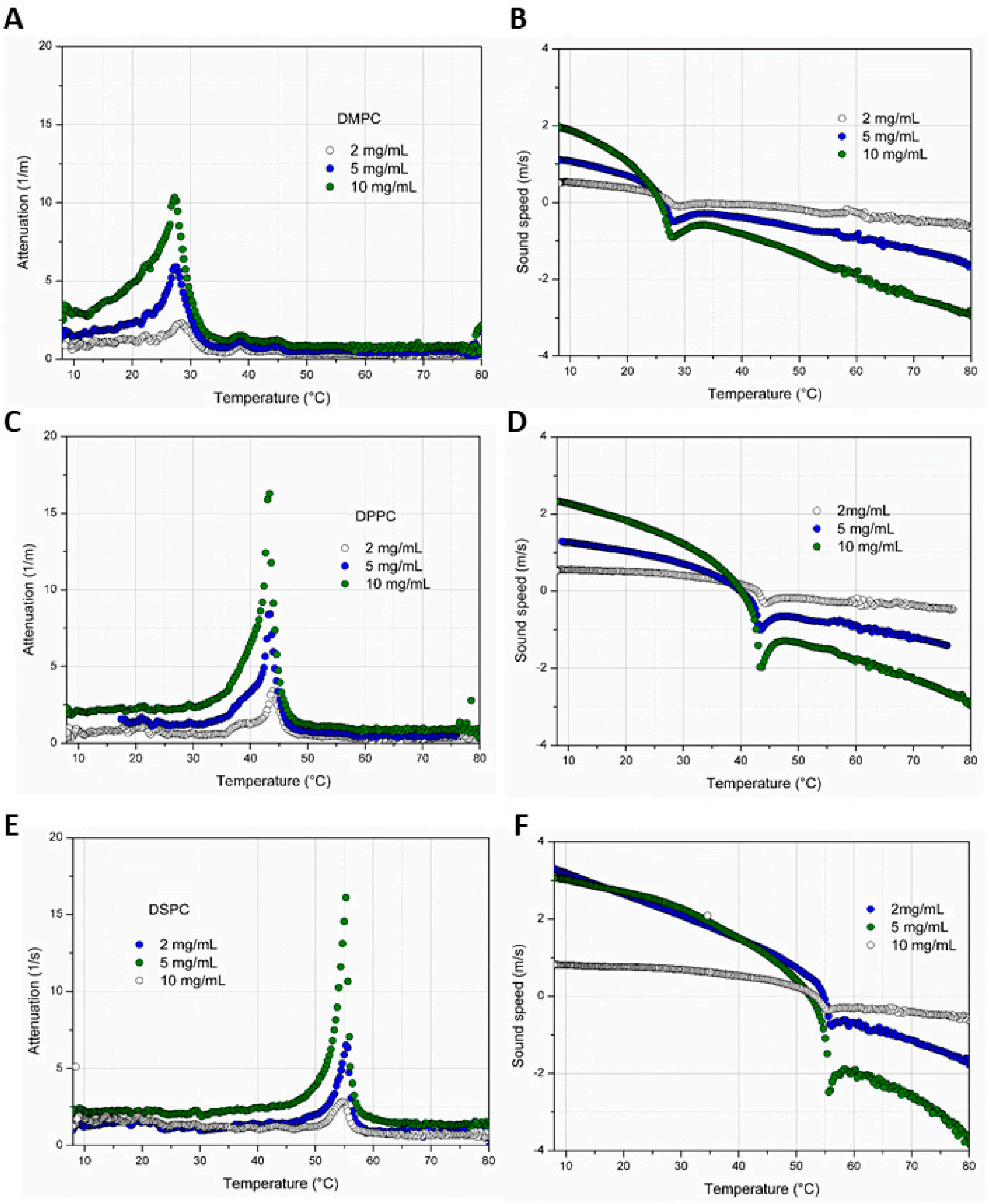

Figure S2. Variation of sound speed and attenuation parameters over temperature for DMPC (**A** and **B**), DPPC (**C** and **D**) and DSPC (**E** and **F**) at different concentrations (2 mg/mL, 5 mg/mL and 10 mg/mL).

Table S2. Thermodynamic parameters (temperature and enthalpy) of the sol-gel transition of liposomes composed of pure phospholipids (DMPC, DPPC, DSPC) at the concentration of 10 mg/mL.

	Microcalorimetry			HR-US	
				Attenuation (1/m)	Sound Speed (m/s)
	Peak (°C)	Onset (°C)	Enthalpy (J/ g of solution)	Transition temperature (°C)	
DMPC 2 mg/mL	24.45 ± 0.04	22.95 ± 0.09	0.056 ± 0.120	24.86 ± 0.07	25.56 ± 0.36
DMPC 5 mg/mL	24.69 ± 0.01	23.29 ± 0.04	0.144 ± 0.006	24.01 ± 0.03	24.01 ± 0.03
DPPC 2 mg/mL	42.06 ± 0.09	40.82 ± 0.01	0.094 ± 0.020	41.41 ± 0.03	42.53 ± 0.31
DPPC 5 mg/mL	41.64 ± 0.01	40.44 ± 0.09	0.211 ± 0.009	41.31 ± 0.19	40.89 ± 0.41
DSPC 2 mg/mL	54.71 ± 0.03	53.24 ± 0.09	0.119 ± 0.009	54.89 ± 0.47	54.89 ± 0.47
DSPC 5 mg/mL	54.60 ± 0.05	53.70 ± 0.02	0.244 ± 0.008	53.14 ± 0.03	54.50 ± 0.15

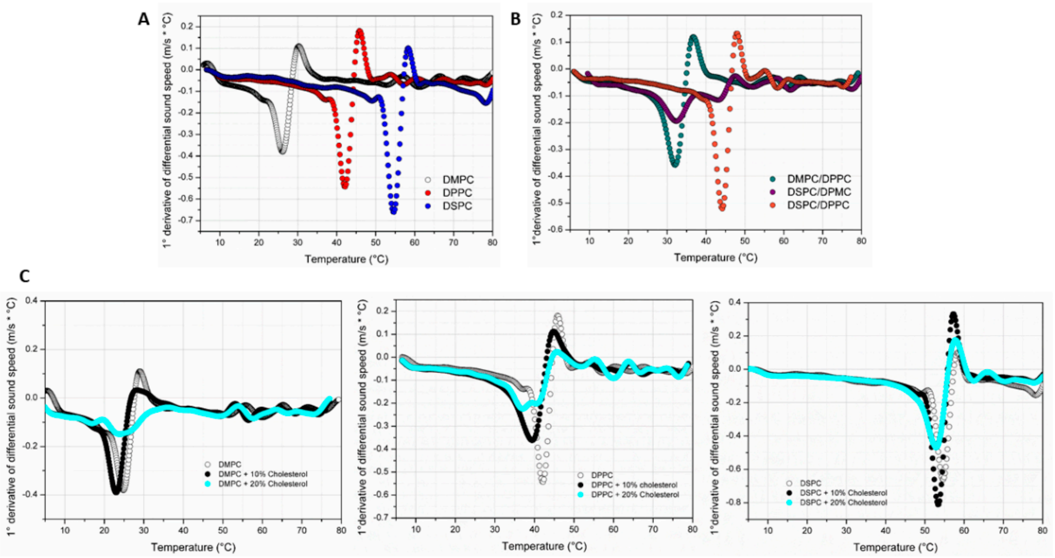

Figure S3. First derivative of sound speed over temperature for pure phospholipids (DMPC, DPPC, DSPC) at a concentration of 10 mg/mL (**A**), for binary mixture (1:1) of phospholipids (DMPC/DPPC, DPPC/DSPC and DMPC/DSPC) (**B**) and for DMPC, DPPC and DSPC in the presence of different percentages of cholesterol (0%, 10% and 20% with the respect to moles of phospholipids) (**C**).

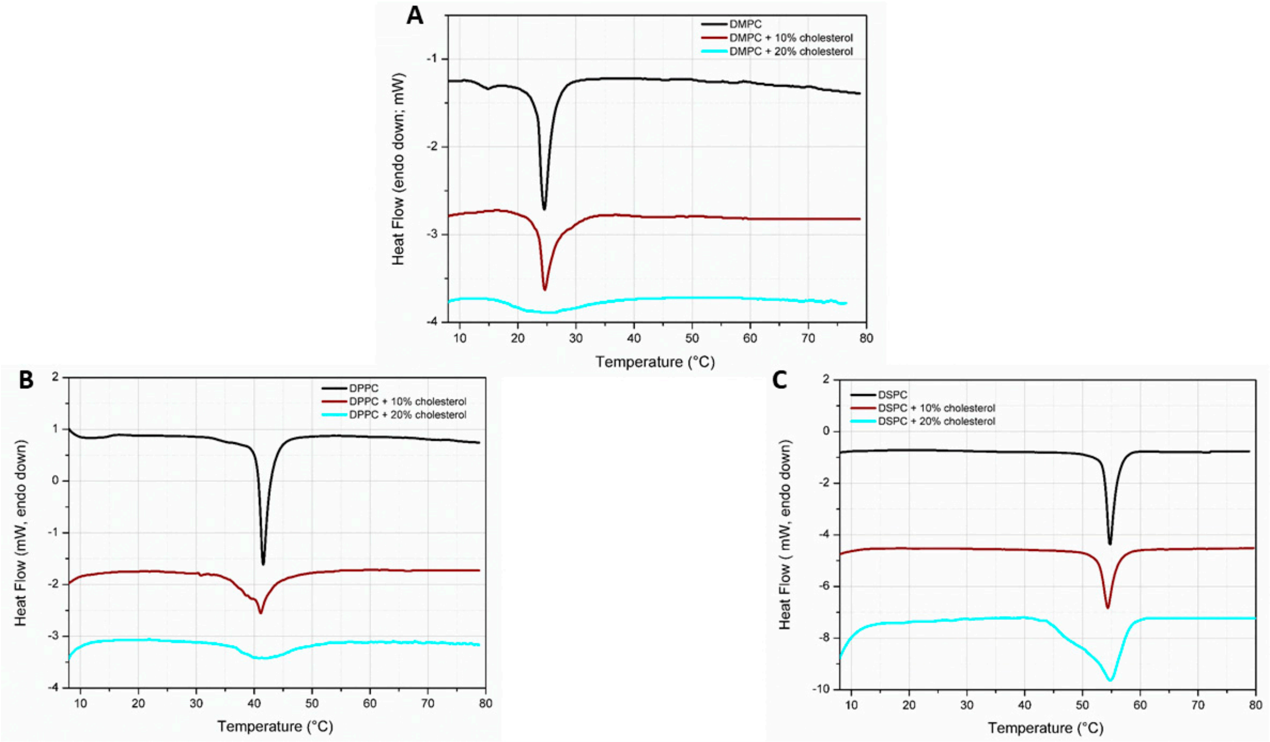

Figure S4. mDSC traces for DMPC (**A**), DPPC (**B**) and DSPC (**C**) in the presence of different percentages of cholesterol (0%, 10% and 20% with the respect to moles of phospholipids).

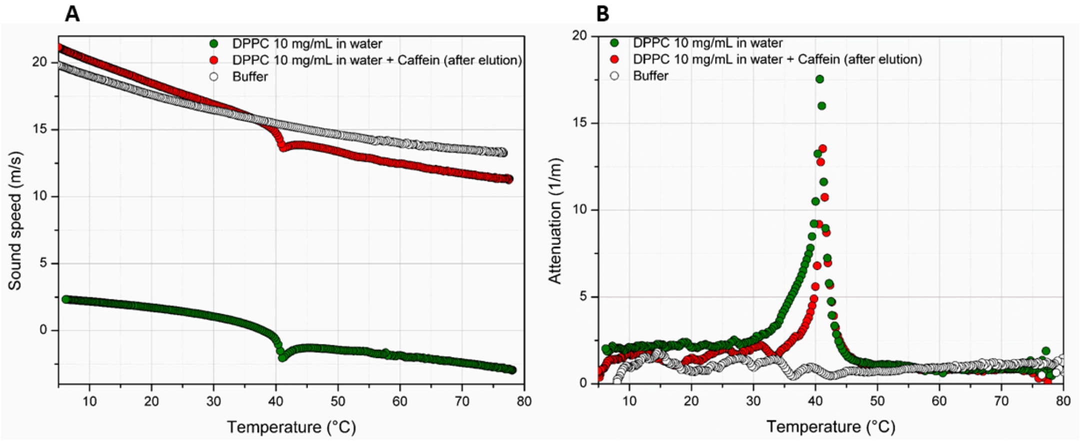

Figure S5. Variation of sound speed (**A**) and attenuation (**B**) parameters over temperatures for DPPC 10 mg/mL, DPPC 10 mg/mL + Caffein (after elution) prepared in water in comparison with the signal obtained by analysing the 50 mM phosphate buffer pH 7.5. All traces were obtained using ultrapure water as reference media for HR-US.
